# HPV disease transmission protection and control

**DOI:** 10.15698/mic2016.09.530

**Published:** 2016-09-05

**Authors:** Neil D. Christensen

**Affiliations:** 1 The Jake Gittlen Laboratories for Cancer Research, Penn State College of Medicine, 500 University Drive, Hershey, PA 17033, USA.

**Keywords:** HPV, animal papillomaviruses, pathogenesis, vaccines, immunotherapy, viral oncogenesis, codon modification

## Abstract

Human papillomaviruses (HPVs) represent a large collection of viral types associated with significant clinical disease of cutaneous and mucosal epithelium. HPV-associated cancers are found in anogenital and oral mucosa, and at various cutaneous sites. Papillomaviruses are highly species and tissue restricted, and these viruses display both mucosotropic, cutaneotropic or dual tropism for epithelial tissues. A subset of HPV types, predominantly mucosal, are also oncogenic and cancers with these HPV types account for more than 200,000 deaths world-wide. Host control of HPV infections requires both innate and adaptive immunity, but the viruses have developed strategies to escape immune detection. Viral proteins can disrupt both innate pathogen-sensing pathways and T-cell based recognition and subsequent destruction of infected tissues. Current treatments to manage HPV infections include mostly ablative strategies in which recurrences are common and only active disease is treated. Although much is known about the papillomavirus life cycle, viral protein functions, and immune responsiveness, we still lack knowledge in a number of key areas of PV biology including tissue tropism, site-specific cancer progression, codon usage profiles, and what are the best strategies to mount an effective immune response to the carcinogenic stages of PV disease. In this review, disease transmission, protection and control are discussed together with questions related to areas in PV biology that will continue to provide productive opportunities of discovery and to further our understanding of this diverse set of human viral pathogens.

## INTRODUCTION

Papillomaviruses are an ancient group of viruses exquisitely adapted to their hosts in a tissue and species-restricted manner (reviewed in 1,2). The human papillomaviruses (HPVs) are responsible for significant morbidity and mortality in the form of various epithelial infections and cancers of skin, anogenital and oral sites (reviewed in 3,4,5,6,7). Classification and evolutionary analyses of sequenced genomes suggest that expansion of new PV types from a primordial type began with the appearance of hair and skin glands in ancestral mammals over 200 million years ago 8. Today, species-specific PVs can be found in most mammals, birds and in several reptiles such as the chelonians and snakes 9. In humans, over 150 types have been fully sequenced 10, with another 200 different HPV genotypes partially sequenced and many more likely yet to be discovered 11. HPV infections are both ubiquitous and common in humans and it is fortunate for us all that most of these infections are benign, clinically asymptomatic and controlled by host adaptive immunity. Currently, approximately 15 different HPV types labeled as high-risk HPV (hrHPV) have been clearly associated with epithelial cancers 12. An additional limited number of types are suspected of having carcinogenic potential 12,13. When viewed collectively, all papillomaviruses share several conserved features including:

a. A small double-stranded DNA genome of around 8Kb, in which numerous viral RNA species are transcribed from one strand only, many of which are represented as multiple spliced transcripts 10.

b. A region containing early genes (usually represented by 5-6 proteins).

c. A region containing two late genes coding for the capsid proteins.

d. A non-coding region containing regulatory elements and a replication origin.

Current control of HPV infections is focused on preventive strategies via induction of neutralizing antibodies; by various ablative strategies for lesion removal, and attempts to activate antigen-specific cell-mediated immunity (reviewed in 14). Questions arise as to why a limited number of these many HPV types progress to malignancy. A combination of immune escape strategies and oncogenic potential of these select types seems the most likely scenario. Many HPV types produce minimal disease and can be best characterized as commensal flora of the skin and mucosa 15. Another important issue related to host control of HPV infections is whether the infectious HPV “load” present in almost all members of the population generates a mild immune-tolerized state due to the commonness of these viral infections. An alternative hypothesis is that these viruses are predominantly immune “invisible” due to viral immune escape mechanisms and lack of inflammatory events *in situ*.

Despite current prophylactic vaccines and other treatment strategies, these viruses continue to be a significant health hazard and also a fascinating group of viruses to whet our appetite for new knowledge on keratinocyte biology, viral oncogenesis, viral tissue restriction and viral evolution. In this review we present an overview of PV biology and propose a series of questions that provide a basis for discussion of some areas of interest that continue to represent important gaps in our knowledge in the HPV research field.

## ETIOLOGY, TRANSMISSION AND PROTECTION

Questions relevant to this section:

*1. Have we found “all” the different HPV types? What would be the predicted number based on current data sets and extrapolation, and are new HPV types continuing to evolve?*

*2. Have we identified all viral proteins/products produced by papillomaviruses during their life cycle? Are there any viral miRNA species yet to be discovered?*

*3. How do new types appear over evolutionary time?*

*4. At what age do infants become infected with HPV? Is there in utero transmission?*

*5. How does the adaptive immune system detect HPV infections in the absence of inflammation?*

*6. Are there sites of infections in patients that show or develop differential immune privilege that allow for increased localized persistence?*

*7. What innate immune escape strategies utilized by PVs are yet to be discovered?*

*8. Will vaccination against a restricted set of HPV types lead to replacement with vaccine-unrelated HPV types? Will vaccines accelerate evolutionary changes in vaccine-matched HPV types or elimination of these types?*

HPV types currently number more than 150 genotypes that have been fully sequenced 10. Papillomavirus researchers have settled on a definition of an HPV type in which a 10% difference in the base sequence of the L1 gene with all existing HPV types is required to define a new type 9,16. Equivalent classification systems can also be developed from sequence comparisons of the viral E6 oncogene, although, interestingly, these methods do not always yield congruent results 17. Intriguingly, there appear to be still many new HPV types yet to be detected as demonstrated by recent analyses on tissue samples using deep-sequencing techniques 11. These new HPV types await further characterization and full sequencing. Classification of papillomaviruses 10 show many different clades or groupings of PVs in which the HPVs are segregated into 3 main groups including Alphapapillomaviruses (mostly mucosotropic), Betapapillomaviruses (mostly cutaneous) and Gammapapillomaviruses (HPVs that include types that can be found in both cutaneous and mucosal sites). When viewed collectively, there remain additional HPV types in other subgroups that are genetically aligned with different animal papillomavirus types.

**Figure 1:**
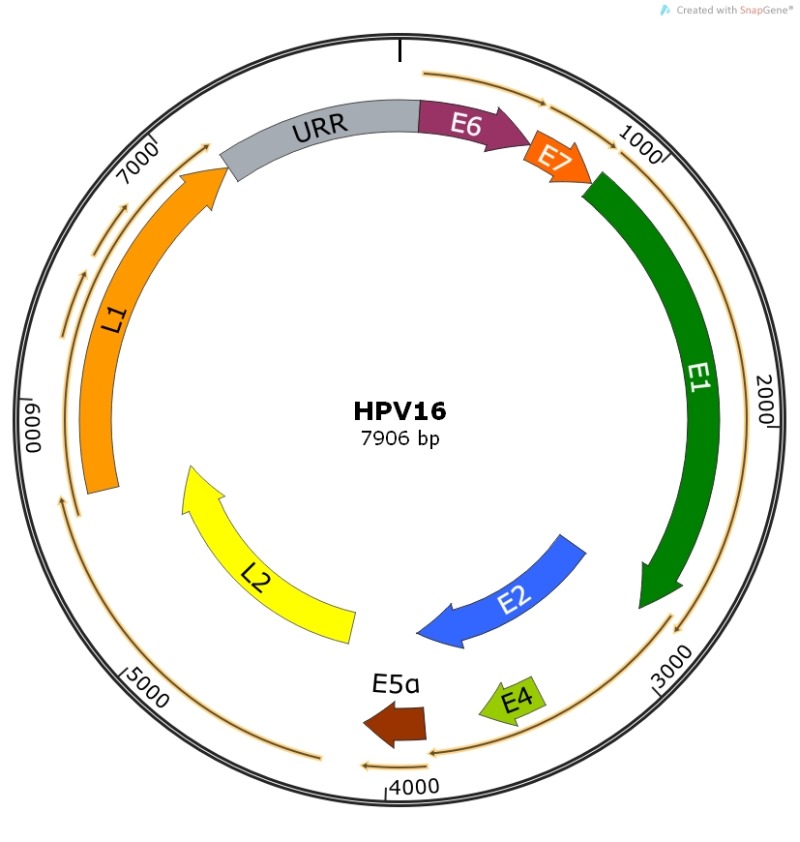
FIGURE 1: HPV16 genome showing viral open reading frames coding for known viral proteins. The Upstream Regulatory Region (URR) is shown in grey. Map constructed using SnapGene software.

PV genomes are approximately 8000 bps of double-stranded DNA with ORFs coding for early (E) and late (L) proteins. ORFs have been identified from the coding sequence for E1, E2, E4, E5, E6 and E7 early proteins and L1 and L2 late proteins (Table 1, Figure 1). Confirmation as to whether additional viral proteins are present (or confirmation that we have now identified “all” PV proteins) are challenging experiments to conduct yet several potential reading frames (e.g. E3, E9, E10, L3) remain unstudied and therefore are assumed to be non-functional and/or non-essential. Spliced viral RNA species have been mapped and “new” proteins (e.g. E8^E2) have been confirmed by studies in an animal papillomavirus model 18 which is amenable to mutational studies. This new protein was later confirmed to be functional in most HPV types 19. Given the smallness of the E5 ORF and the redundant functional activities of hydrophobic E5-like proteins 20 we should be encouraged to continue more systematic analysis of other small uncharacterized ORFs and understudied spliced proteins (e.g. E6^E7 21) in PV genomes. It is well-recognized in other virus systems that there are many viral proteins that operate as host restriction factors and/or immune function modulators that can be confirmed only by studies using *in vivo* models.

**Table 1:** Papillomavirus proteins and functions.

**Viral protein**	**Function**	**Host protein interactions**	**References ** **(mostly reviews)**
E1	Helicase, interacts with PV E2.	DNA polymerase alpha and RPA.	22
E2	Transcriptional repressor, transcriptional enhancer.	Brd4, TopBP1.	23 24
E4	Association with the cellular keratin network. A likely role in virus release and transmission.	Cellular keratins?	25
E5	Hydrophobic protein that can dimerize growth-factor receptors, deregulate the autophagic process, modulate epithelial-mesenchymal transition and reduce some host microRNA.	Vacuolar ATPase, PDGF, zinc transporter ZnT1, protocadherin 1 (PCDH1), and AHNAK/desmoyokin, YIP1 family member 4, calpactin I.	20 26 27 28 29
E6	Viral oncogene. A negative regulator of UV-DNA damage repair (betaPVs).	p53 (alphaPVs), E6AP (alphaPVs), histone acetyltransferase p300/CBP (betaPVs), MAML1 (betaPVs), and proteins containing PDZ domains. 153 cellular proteins.	30 31 32
E7	Viral oncogene.	pRB, CENP-C.	33
L1	Major capsid protein.	HSPG, L332, cyclophylins.	34,35,36
L2	Minor capsid protein.	PATZ, PLINP, PMSP, dynein.	37 38
E8	E5-like protein in several animal papillomaviruses. Not similar to the E8 protein fragment of many HPVs.	Zinc transporter ZnT1, protocadherin 1 (PCDH1), and AHNAK/desmoyokin.	39
E8^E2C; E9^E2C; E8^E1; E1M^E2C E6^E7	Transcriptional repressor. (E9^E2C represents the equivalent spliced product in the CRPV genome).	NCoR1 and TBLR1.	19 40 41 21

HPV infections are believed to occur following wounding of epithelium and subsequent infectious virion access to basal epithelial cells and basement membrane components of the epithelium (reviewed in 1). Enhanced infection following wounding has been confirmed experimentally in preclinical models 42,43. Current diagrams often depict virions entering a breach in the epithelium whereby free virions reach the basal cells and initiate infections. PV-associated lesions are then maintained via persistence of viral-infected basal cells and the lesions increase in mass via replication of infected cells coupled with epithelial differentiation. Vertical maturation of infected keratinocytes completes the virus life cycle culminating in virion assembly in the upper layers of the wart. Natural transmission of cutaneous infections likely involves physical contact of the upper keratinized wart with normal skin generating microabrasions allowing virus-containing squames to be shed into the wounded site. Environmental contact between virus-laden shed squames and skin surface wounds are also a likely transmission mechanism. Virion release from the squames may include a combination of keratin filament disassembly events involving viral E4 proteins 25, and host/microbial proteases 44 with subsequent release of the cell-free virions into the wounded site. Mucosal infections are also believed to occur following mechanical wounding during sexual intercourse for vaginal and anal infections.

A question arises as to whether virions can access basal cells via a retro-transport mechanism in areas of epithelial “conflict” at what are known as transition zones located in the cervix and anal canal 45. Cell culture experiments have demonstrated that virions can bind to cellular filopodia and subsequently be retro-transported significant distances 46 suggesting possible cell-to-cell or cell-to-ECM (extracellular matrix) transfer 34,47 that would bypass the need for direct wounding. Other viruses use a similar strategy of cell-cell transfer and epithelial basement membrane interactions (reviewed in 44). The transition zone sites 48 are particularly vulnerable to persistent HPV infections that can lead to malignant progression 45,49. A mechanism to describe the selectivity and exclusivity of entry of HPV virions into these unique sites is not easy to reconcile with the general concept of transmission via site-specific mechanical damage during sexual intercourse.

### Natural protection and control of HPV infections

Papillomaviruses have developed a variety of strategies to escape host innate and adaptive immunity (reviewed in 50,51). A central tenet for the failure of immune control and detection is the lack of inflammatory events during the various stages of infection. PV infections are confined to epithelial tissues and are highly localized. Several viral proteins are involved in immune escape (Table 1), including: (i) E5, which can down-regulate MHC Class I and other key molecules in the antigen-presentation pathways 52,53; (ii) E6 and E7, which can suppress host interferon pathways 54,55,56, activate the DNA-damage pathways 57,58,59, and induce immune suppression via activation of suppressive cytokines and Tregs 60,61.

Clear evidence for immune control stems from studies on preclinical models 62 and immunosuppressed patients 63,64,65. In the preclinical cottontail rabbit PV (CRPV) model, two natural CRPV variants exist in which one variant is poorly immunogenic and produces persistent infections whereas the second variant is immunogenic and easily cleared by host immunity 66. Several amino acid differences in the E6 protein alone altered the persistor strain to an immunogenic or regressive phenotype 67. Interestingly, when the rabbits were immunosuppressed prior to infection with the regressive variant then subsequently released from immunosuppression, persistent lesions were observed in some (but not all) animals 68. These preclinical results support similar observations in immunosuppressed patients where persistent HPV infections are prevalent and can expand to clinical disease during immunosuppression 63. The implications of these studies are that HPV infections may develop into persistent infections following immune depression arising via temporary environmental stresses. Thus, under normal immunocompetent conditions these infections would have been cleared effectively.

#### Host restriction factors

The exquisite tissue restriction of some HPVs has attracted recent interest in the “new” fields of viral-host interactions in which various host restriction factors influence virus life cycles 69. Some recent observations in HPV *in vitro* models indicate roles for APOBEC3 family members 70,71, DNA-damage repair (DDR) pathways 57,58,59, IFN-kappa 72,73, IFI16 74, TLR9 75,76 and IL1β 77,78 as host restriction factors or pathways that the virus must overcome. Recent studies on the DNA sensor, IFI16, suggested that the proposed editing of HPV in cervical cancer may be linked to HPV-mediated induction of a human APOBEC3-dependent intrinsic host defense mechanism 74. DDR pathway activation and suppression occurs in HPV replication and carcinogenesis mediated by viral E1 and E2 (repression) and viral E6 and E7 (activation) respectively 59.

New observations on the role of HPV in autophagy also demonstrate host-mediated control pathways disrupted by HPV 79,80,81. Finally, there are potential impacts on HPV infection via host microRNA 82,83. Recent studies have begun to search for various miRNA species as markers for HPV-associated cervical and oral cancers and precancers (reviewed in 84,85). Collectively, these areas of research will continue to provide a fruitful avenue of new observations in improving our understanding of host control of HPV infection and carcinogenesis.

#### Innate and adaptive immune modulators

Potential innate and adaptive immune control of PV infections must be thwarted by these viruses in order to complete their life cycle. At the same time, the virus must use many host factors and the host replication machinery for completion of their life cycle. Studies show that central adaptive immune control of HPV infections is by type-1 interferon (IFN) and tumor necrosis factor (TNF)-α cytokine-producing T cells 86. Down-regulation of interferon pathways is a common virus escape mechanism, and HPVs can accomplish innate immune evasion by augmenting the expression of interferon-related developmental regulator 1 (IFRD1) in an EGFR-dependent manner 87. In addition, the E7 protein of hrHPV has been shown to bind HDAC1 and prevent acetylation of histones, thereby suppressing signaling from the innate immune sensor, TLR9 75. Codon usage has also been hypothesized to alter immune detection and responsiveness to different HPV classes 88. A better understanding of these various immune escape strategies will be needed to improve immunotherapeutic approaches to HPV management.

## PATHOLOGY/SYMPTOMATOLOGY 

Questions relevant to this section:

*1. Why do infections with some HPV types manifest only as asymptomatic disease whereas others show active clinical disease?*

*2. Why do infections by different HPV types progress to cancer whereas others do not?*

*3. Do HPV16-associated cancers at different anatomical locations have a similar pathology, etiology, and progression rate?*

*4. Are HPV-associated cancers less immunogenic (or have differences in immune escape mechanisms) than precancerous or primary benign HPV infections of the same HPV type?*

*5. Does the detection of HPV viral DNA correlate 100% with active clinical disease?*

HPV infections present as epithelial lesions that are localized to cutaneous or mucosal sites. All cutaneous tissues are susceptible to HPV infections although mucosal infections are mostly confined to the anogenital and oral cavities as well as laryngeal epithelium. Other less common mucosal sites include bladder, conjunctiva and lung epithelium. The infections range from asymptomatic infections (particularly from the beta- gammapapillomaviruses) to small epithelial lesions to very large cutaneous lesions to large cancerous lesions. In general, most infections are small in size, and present with minimal evidence of infection (no fever, rash, itchiness or any other signs of discomfort).

Many HPV infections are confirmed by sensitive DNA detection methods rather than via histological or *in situ* analyses. Those HPV types that produce asymptomatic disease are challenging to locate *in situ*. That these asymptomatic infections are true infections that complete the virus life cycle and release infectious virions is absolutely confirmed by the existence and persistence of these many HPV types in patient populations.

One HPV type (HPV16) stands out as highly associated with cancers at several different anatomical locations (cervix, penis, anus, oropharynx, and other rare sites such as the esophagus 89 and bladder 90,91). These lesions have been examined extensively for various diagnostic markers and yet the reasons for differential progression rates and susceptibility of the various sites remain unclear. We also do not yet know whether the innate and adaptive immune systems respond differently to HPV infections at these different anatomical locations. Another unknown is the potential differential immune response to oncogenic HPV infections at the early benign/precancerous stage versus the later carcinomatous stage. There may be enhanced immune escape mechanisms in play in the latter cases that allow a site-specific cancer to eventuate. Also complicating the issue is that there are now a number of different HPV16 subtypes and mutants with differing levels of infection and progression rates in different patient populations 92.

The primary oncogenic potential of HPV types resides in 3 major viral proteins, E5, E6 and E7 20,93,94,95. For hrHPV types in the alphapapillomavirus group, extensive *in vitro *studies and transgenic animal models have confirmed the oncogenic potential of E6 and E7 proteins 96,97,98,99,100,101. Oncogenic activities of the E6 and E7 proteins includes p53 sequestration, pRB binding, interference with DNA damage response pathways, disruption of cell cycle and cell division pathways, and immune evasion (reviewed in 5,51,102). An oncogenic role for some betapapillomaviruses such as HPV5 and HPV8 includes important co-factors such as UV-damage and include p53 modulation 65,103. Untreated HPV-associated cancers can progress to malignant cancers that are locally invasive, difficult to treat, and often lead to death of the host. New pathways for transformation are continuing to be identified (e.g. role of autophagy 104). There is a generalized assumption that malignancies in different anatomical locations initiated by the same HPV type are similar mechanistically. As mentioned previously, we are likely to find both unifying and unique molecular and cellular events when studying HPV16 (and their variants) infections of the cervix, vagina, penis, anus and oral cavity.

HPV types that infect skin show different histopathology when compared with HPV types that infect the mucosa. Notably, differences in the impact of the viral E4 protein expression lead to phenotypic uniqueness of infections by alpha- versus betapapillomaviruses 105. The high production of the E4 protein in the lesions can be used as a diagnostic marker for various HPV diseases 106,107. New biomarkers are continuing to be discovered and continue to be needed to assist clinical diagnoses, assess treatment outcomes, and to inform physicians as to the appropriate management and treatment of HPV disease.

## EPIDEMIOLOGY, INCIDENCE AND PREVALENCE

Questions relevant to this section:

*1. Have we discovered all the risk-factors associated with HPV progression and cancers?*

*2. What viral and host factors (gender, race, age and anatomical site) are associated with a potential differential susceptibility to HPV infections.*

*3. Given the commensal nature of some skin and mucosotropic HPV and their prevalence, do these commensal infections provide a potential localized immune tolerance for subsequent infection by the more pathogenic HPVs thereby reducing the initial host immune response to these latter types?*

*4. Is there competition or synergistic interactions between different HPV types during a co-infection?*

*5. Do asymptomatic HPV infections trigger protective homeostatic host immunity to these and other common skin microbes? Do other microbes at skin and mucosal sites provide immune “cover” for many asymptomatic HPV infections?*

*6. Is there a correlation between codon usage profiles and tissue tropism for HPV types?*

HPVs are one of the most ubiquitous viral infections with over 300 viral types that infect most cutaneous and many mucosal tissues. Most types are associated with minimal disease and can be described as part of the commensal fauna of these tissues. Numerous epidemiological and molecular studies on cervical cancer demonstrate that hrHPV are associated with up to 99% with cervical cancers 3,14. This near 100% association between an infectious agent and a particular cancer is unique to all infectious causes of cancer 108. Nevertheless, HPVs are considered a necessary but not sufficient cause of HPV-associated cancers 4,109,110,111 and a number of co-factors and risk-factors have been documented 112. These co-factors are represented by (i) coincident infectious agents such as EBV, HSV-2, HIV and Chlamydia 111,113,114,115,116,117; (ii) environmental factors (various chemical agents, UV light, immune suppression, stress); (iii) behavioral factors (sexual partners, parity and smoking) and (iv) genetic and epigenetic factors (various polymorphisms in HLA molecules, p53 and GST 118,119, the genetic predisposition known as epidermodysplasia verruciformis, and methylation status of the viral genome) (reviewed in 1,120,121). Several studies have indicated that cervical cancer development and progression may be closely associated with a dual-infection with HPV and EBV 4,115. Infiltrating EBV-infected lymphocytes have been detected in cervical lesions containing episomal hrHPV 115. The impact of co-incident chronic inflammation and immune modulation via these infectious co-factors has not been studied mechanistically in preclinical models and warrants further study.

HPV of the betapapillomavirus group such as HPV5 and HPV8 are associated with rare incidences of skin cancers, and these and other members of this family are considered to also play a role in non-melanoma skin cancers 30,109,122. The oncogenic potential of members of the betapapillomaviruses are hypothesized to require co-factors including UV light and p53 polymorphisms 123. The roles of the viral oncogenes in these instances include activation of telomerase and extension of cell life span via E6 124, and a potential cancer correlation with E6 and polymorphisms in p53 103,123.

### Tissue tropism, latency and viral reservoirs

There is an exquisite interplay between HPV persistence, tissue tropism and the host innate and adaptive immune response. The keratinocyte is the exclusive host cell of HPVs, and has significant potential to mount both anti-viral responses to PV infections as well as activate adaptive immunity. The tissue tropism introduces the concept of micro-environments in which the host keratinocytes and local immune monitoring are not necessarily identical at different anatomical sites. Possible differences in immune monitoring as well as differences in the natural microbiome in oral and anogenital mucosa also provide mechanistic explanations for HPV tissue tropism 45,49. Epithelial stem cells have attracted interest as a primary source of susceptible cells for initial infection 45,48. The epithelial transition zone may also act as a stem cell niche and thus represents a key location for cellular transformation by accumulated genetic mutations or viral transformation resulting in tumor formation 48. Several proteins that are induced during wounding are expressed specifically within transition zones, and/or on epithelial stem cells, and may correlate or contribute to HPV-associated transformation 125,126,127.

The exquisite and regional tissue restriction of some HPV types is well-illustrated by such examples as the association of HPV7 with meat-handlers 128. HPV7 appears to be sufficiently common, but the clinical disease of hand warts appears to be correlated with the unique occupational damage to the hands of these workers rather than via person-to-person transmission of HPV7 129. Other skin-tropic HPV types are more common on feet and hands (HPV1/2/4/60/63). Clearly, some HPV types can discriminate at the level of regional tissue sites that the investigator would predict to be tissue-site equivalent. Cellular and molecular differences in keratinocytes are a plausible explanation for these regional specificities, as is the potential differences in the local microbiome and differential immune responsiveness *in situ*. This phenomenon represents another fruitful area of research for the papillomavirus community. Note too that some HPV types show little tissue restriction and can be found at both mucosal and cutaneous sites (e.g. members of the beta- and gammapapillomaviruses 130).

The concept of viral latency in papillomaviruses has also raised significant interest in the research community 15,131. Strong circumstantial evidence exists with patients that undergo immunosuppression either by infection (e.g. HIV) or for organ transplantation. Again, the existence of viral DNA and RNA at low levels in clinically normal tissues following immune clearance is confirmed in preclinical models 132. Additional studies to determine the viral mRNA profile would improve our understanding of HPV latency and help determine whether the viral program is reduced in content via immune (and/or innate immune) monitoring or whether distinct components of the viral life cycle are silenced in keeping with other viruses known to have classical latent phenotypes (such as herpesviruses).

The concept of viral reservoirs for secondary infections has attracted limited coverage in the literature 133. The consensus view is that viral reservoirs include multiple localized infections that may be either clinically active or asymptomatic but which continue to shed infectious virions leading to self-inoculation. Such scenarios may provide a source of virus that can set up secondary infections at transition zones, or sites that are known to progress to malignancies. Recently, non-genital sources of virus present under fingernails has been recorded that could potentially provide an alternate reservoir for future infection although the investigators concluded that this method of transmission is unlikely 134.

Codon usage profiles of papillomavirus proteins show striking differences between the papillomavirus groups 88, between viral proteins 135,136 and disease phenotypes 88. These studies highlight another intriguing mechanism by which papillomavirus tissue tropism could be influenced, and such concepts can be directly assessed using preclinical papillomavirus models and mutational analyses 137.

### Tropism and tissue site selectivity; lessons learned from a mouse PV (MmuPV-1) model.

**Figure 2:**
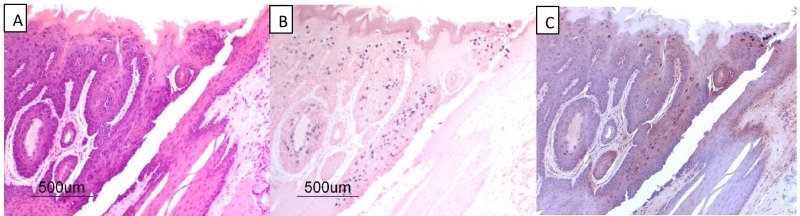
FIGURE 2: MmuPV1 secondary infections of the mouse oral cavity. **(A)** (H&E), **(B)** (*in situ* hybridization using MmuPV1 DNA probe) and **(C)** (immunohistochemical staining using a monoclonal antibody to MmuPV-1 L1 protein) detecting an infection localized to the base of the tongue at the circumvallate papilla. **(A)**, **(B)** and **(C)** are successive 4 μm sections from formalin-fixed paraffin-embedded tissues from athymic mice. One of several examples of MmuPV-1 infection of the circumvallate papilla of the mouse tongue.

A new mouse PV model has recently been described that infects cutaneous 138 and mucosal tissues 139. Although this virus type is clearly genetically dissimilar to the more well-studied alphapapillomaviruses, the tissue restriction of HPV for human tissues prevents mechanistic study of these viruses *in vivo* and thus preclinical models provide insights into the tissue selectivity of papillomaviruses. We have observed that when the oral cavities of mice are secondarily exposed to MmuPV-1 infectious virions without experimental wounding, select sites in the oral cavity (circumvallate papilla, base of tongue) become preferentially infected despite the observation that most oral mucosa is susceptible to this virus (Figure 2). These studies mimic to some extent, the pre-selection of oral HPV-associated cancers that are confined to the base of tongue, tonsil and oropharynx 5. We do not yet understand why certain sites in the oral cavity are more vulnerable to HPV malignancies than other sites. Some new observations regarding stem cells in skin hair follicles, cervix and anal epithelial transition zones and tonsillar crypts are suggestive of such cells being prime targets for HPV infection, and sites that preferentially progress towards malignancy 45,49.

## TREATMENT AND CURABILITY

Questions relevant to this section:

*1. Why do some patients clear their HPV infections and others do not? Are there genetic, epigenetic and/or environmental components to these different outcomes?*

*2. Why do many patients clear infections against some HPV types but not others?*

*3. Do pre-existing co-infections with other infectious agents increase or decrease susceptibility to and persistence of HPV infection?*

*4. Does immune clearance of HPV infections lead to sterilization of the infection or immune monitoring of a subclinical persistent infection?*

*5. Why are there so many treatment failures when clinical “cures” appear to have been achieved with various different strategies?*

*6. Can we develop successful T-cell based immunity to existing HPV disease that can control/clear persistent disease and cancer? Are there additional neo-antigens (in cells with integrated viral DNA) such as host-viral fusion proteins that could serve as potential CD8 T-cell epitopes?*

Asymptomatic HPV disease remains untreated as a default. Active clinical disease is diagnosed by both sensitive viral DNA detection methods, and histology or cytology. Monitoring programs (e.g. PAP test) are used to determine the timing and severity of intervention. The PAP smear test examines cells collected by lavage from vaginal sites and cells are examined for histological abnormalities, for viral DNA or for other diagnostic indicators such as increased p16INK4a immunostaining, acid phosphatase positivity, p53, p63, various host microRNA and other additional novel markers 82,85,118,121,126,140,141,142,143,144,145,146. Biopsy material is examined for histological markers and graded using a CIN classification (stages I to IV) or via the Bethesda system for high or low grade dysplasia. Ablative or topical treatments are mostly on an *ad hoc* basis of treatment of visible lesions seen by colposcopy or acid-white staining.

### Therapeutic interventions

Three prophylactic vaccines are currently approved for control of several high-risk HPV types, and two low-risk types commonly found in genital warts. These include Gardasil (HPV6, 11, 16 and 18), Cervarix (HPV16, 18) and Gardasil9 (HPV6, 11, 16, 18, 31, 35, 45, 52, 58) 147,148,149. The vaccines use virus-like particles for the designated HPV types and trigger high titers of type-specific neutralizing antibodies and excellent protection against vaccine-related HPV types 150,151,152. Some limited cross-protection against vaccine-related types has been observed in clinical trials 153. Second-generation vaccines have increased the number of HPV types in the vaccine (e.g. Gardasil9) and are now considering L2-based vaccines that are more broadly cross-protective 154,155.

Therapeutic vaccines based on T-cell responses to virus-infected tissues have been extensively tested in preclinical models, but have enjoyed much less success in clinical trials. Nevertheless, there have been recent encouraging results using long peptide vaccines from HPV16 E6 and E7 for therapeutic T-cell based regression of vaginal intraepithelial infections 156,157. Adoptive transfer of HPV16-reactive T-cells also shows promise against cervical cancer in an initial clinical trial 158. As we go forward with new and improved immunotherapeutic approaches to manage HPV-associated cancers, there are several challenges to our knowledge base that require further studies. Some examples follow:

a. We need to better understand the various immune suppressive events that occur *in situ* in HPV-associated cancers that are highly localized 159.

b. We need to design improved strategies to overcome localized T-cell exhaustion and other functional deficiencies that are increasingly being defined in other chronic viral infections 160.

c. We need to better understand and then counteract HPV-induced innate and adaptive immune escape mechanisms to improve T-cell recognition and elimination of HPV infected cells.

d. We need to improve our understanding of T-cell homing to non-immunological tissues (vaginal, anal and oral mucosal epithelium) in order to improve the frequency and effector functions of CD8 T-cells 161.

e. We need to improve therapeutic activation of HPV-specific T-cells and assess other potential non-HPV epitopes arising from mutations in cervical cancer to better design vaccines that develop long-lasting immunity 162,163.

Finally, there may be productive opportunities to explore possible sources of neo-antigens in HPV-containing cancer cells that are represented as novel mutations in host cell proteins that provide new CD4 and CD8 epitopes 164,165,166. Another possible source of neo-antigens may arise in cancer cells with integrated viral genomes that could express hybrid host/viral fusion proteins. Additional sources of neo-antigens may arise from altered expression of viral proteins through the process of defective ribosomal products (DRiPs) 167,168,169.

## MOLECULAR MECHANISMS OF INFECTION

Questions relevant to this section:

*1. Is wounding essential for successful infection of tissues by infectious virions?*

*2. Can HPV infections be established from virions that enter sites of epithelial “conflict” as found in transition zones, hair follicles and tonsillar crypts, without the need for environmental wounding?*

*3. Do all HPV types use the same molecular entry pathway?*

*4. Do different HPV types interact with different host factors during disease persistence and progression?*

*5. Is the mechanism of virion entry different for cutaneous versus mucosal epithelium?*

*6. What are the molecular, genetic and immunological criteria that define tissue-tropism of PVs?*

Papillomaviruses are predominantly epitheliotropic. Only a small number of PV types confined to the deltapapillomavirus group induce fibropapillomas in which both epithelial and fibroblast cells are infected 10. Current dogma proposes that virions enter sites of epithelial damage from wounding. Such wounds expose the basal cells and the basement membrane of the epithelium to virions and the subsequent wound-healing environment is believed to be essential for the establishment and maintenance of PV infections. Wounding may be achieved via mechanical, chemical or biological means. Mechanical damage to skin can occur via cuts, abrasions and UV damage; via intercourse for vaginal and anal mucosa and via eating, smoking and oral cleansing for oral mucosa. Chemical damage can occur via contraceptives, suppositives, oral and vaginal lavage solutions and oxidative damaging agents. Biological damage can occur via co-incident microbial agents such as bacteria, protozoans and fungi that can erode tissues and the basement membrane leading to remodeling and repair of the epithelium 44. Once basal cells are infected, PV genomes proceed through 3 stages of replication that can be summarized as (i) initial amplification within basal keratinocytes,(ii) followed next by a maintenance phase as the infection becomes established, then (iii) a vegetative stage where viral genomes are amplified to high copy and packaged into virions 58.

### *In vitro* models of entry

Molecular events of entry have been studied extensively in cell culture systems (reviewed in 36,170,171,172). Both native virions from natural infections, xenografts and organotypic cultures as well as synthetic particles prepared in 293TT cells 173 have been used for studies on entry kinetics and receptor analyses 174. A general consensus of these studies describes a key role for heparin-sulfate proteoglycans (HSPGs) as a primary binding receptor both *in vitro* 175 and in a pseudovirus infection model of cervicovaginal tissues in mice 43. Variation in responses is seen when using different viral and pseudoviral systems, different cell lines and different methods for preparing reagents and between different viral types 170.

A series of different host proteins are involved in the various early stages of binding and entry. These include surface heparin-sulfate proteoglycans (e.g. syndecans) 35,176, extracellular matrix (ECM) proteins (e.g. Laminin 332) 34, anexin A2 177, cyclophilins 178 and tetraspanins 179. Again, a general consensus model supports the hypothesis that virion binding to HSPGs and ECM components leads to structural changes to the capsid surface allowing exposure of a region of the minor capsid protein to furinase cleavage 180 and subsequent transfer to a secondary receptor for receptor-mediated uptake 181. Various studies have demonstrated both support and contradiction to several of these proposed steps suggesting that there may be different uptake and entry pathways for the different HPV types rather than a unifying single molecular entry pathway 170,182,183.

### *In vivo* models of entry

Several animal preclinical models have been used to assess some features of viral entry *in situ* onto skin and mucosal sites. These studies include mouse cervicovaginal 43 and rabbit skin infection models 184. These studies provide evidence for a role for HSPGs, for virion targeting to the ECM, and for pre-wounding prior to infection 42,43. The studies also clearly indicate that the PV capsid proteins do not account for tissue and species tropism at the level of viral entry into the epithelial cells. Thus, HPV capsids can efficiently deliver papillomavirus genomes into rabbit skin 184 and plasmid genomes into mouse skin and mucosa 43.

## CONCLUSIONS

*Do we have sufficient knowledge about the complex interplay between immune mechanisms of escape, viral carcinogenesis, host innate sensors, transcriptional regulation and codon usage profiles to determine the species and tissue tropism of papillomaviruses?*

In summing up, the PV research community has built an impressive body of knowledge on the biological, clinical and pathological activities of papillomaviruses. Significant challenges remain in the arena of improved treatments for persistent and latent HPV infections and associated cancers. Preclinical PV models will continue to provide opportunities for more mechanistic studies where the virus and host can be manipulated both genetically and pharmacologically to tease apart contributions of the various components of the virus life cycle. Already, these viruses have enriched our knowledge of viral evolution, tumor suppressor function, host restriction factors, innate immunity, codon usage profiles, viral vaccines, immunotherapy, DNA damage response mechanisms, keratinocyte biology and viral tissue tropism. Future studies will allow us to assess the impact of the current prophylactic vaccines on a select set of HPV types and the selection pressures these vaccines impose on viral evolution. Papillomaviruses have provided a resource for a variety of expertise spanning the fields of virology, immunology, pathology, gynecology, evolutionary biology, vaccine manufacture, carcinogenesis and molecular virology. Questions remaining will continue to challenge future researchers as we continue to study these fascinating viruses.
